# Dr. Henry William Brown

**Published:** 1913-04

**Authors:** 


Dr. Henry William Brown, Geneva Medical College, 1867, a surgeon of volunteers during the Civil War, died at Waterloo, Ia., Feb. 27, of organic heart disease, aged 68. (Note.—The Geneva Medical College existed from 1836 to 1872, when it was merged with the Medical Department of Syracuse. As Geneva contributed indirectly to the University of Buffalo, the University of Michigan and other extant colleges, and as there can be comparatively few of its graduates living, we would welcome a historic review of its work.)

